# The transcriptomic landscape of *Magnetospirillum gryphiswaldense* during magnetosome biomineralization

**DOI:** 10.1186/s12864-022-08913-x

**Published:** 2022-10-10

**Authors:** Cornelius N. Riese, Manuel Wittchen, Valérie Jérôme, Ruth Freitag, Tobias Busche, Jörn Kalinowski, Dirk Schüler

**Affiliations:** 1 Department of Microbiology, University of Bayreuth, Bayreuth, Germany; 2 Center for Biotechnology (CeBiTec), University of Bielefeld, Bielefeld, Germany; 3 Chair for Process Biotechnology, University of Bayreuth, Bayreuth, Germany

**Keywords:** *Magnetospirillum*, Magnetosomes, Operons, Promoters, Transcription, Transcriptome

## Abstract

**Background:**

One of the most complex prokaryotic organelles are magnetosomes, which are formed by magnetotactic bacteria as sensors for navigation in the Earth’s magnetic field. In the alphaproteobacterium *Magnetospirillum gryphiswaldense* magnetosomes consist of chains of magnetite crystals (Fe_3_O_4_) that under microoxic to anoxic conditions are biomineralized within membrane vesicles. To form such an intricate structure, the transcription of > 30 specific structural genes clustered within the genomic magnetosome island (MAI) has to be coordinated with the expression of an as-yet unknown number of auxiliary genes encoding several generic metabolic functions. However, their global regulation and transcriptional organization in response to anoxic conditions most favorable for magnetite biomineralization are still unclear.

**Results:**

Here, we compared transcriptional profiles of anaerobically grown magnetosome forming cells with those in which magnetosome biosynthesis has been suppressed by aerobic condition. Using whole transcriptome shotgun sequencing, we found that transcription of about 300 of the > 4300 genes was significantly enhanced during magnetosome formation. About 40 of the top upregulated genes are directly or indirectly linked to aerobic and anaerobic respiration (denitrification) or unknown functions. The *mam* and *mms* gene clusters, specifically controlling magnetosome biosynthesis, were highly transcribed, but constitutively expressed irrespective of the growth condition. By Cappable-sequencing, we show that the transcriptional complexity of both the MAI and the entire genome decreased under anaerobic conditions optimal for magnetosome formation. In addition, predominant promoter structures were highly similar to sigma factor σ^70^ dependent promoters in other Alphaproteobacteria.

**Conclusions:**

Our transcriptome-wide analysis revealed that magnetite biomineralization relies on a complex interplay between generic metabolic processes such as aerobic and anaerobic respiration, cellular redox control, and the biosynthesis of specific magnetosome structures. In addition, we provide insights into global regulatory features that have remained uncharacterized in the widely studied model organism *M. gryphiswaldense*, including a comprehensive dataset of newly annotated transcription start sites and genome-wide operon detection as a community resource (GEO Series accession number GSE197098).

**Supplementary Information:**

The online version contains supplementary material available at 10.1186/s12864-022-08913-x.

## Background

Magnetosomes, which are formed by magnetotactic bacteria (MTB) as sensors for geomagnetic navigation in their aquatic habitat, represent an example for one of the most complex organelles found in prokaryotic cells [1–3]. Their unprecedented magnetic properties make bacterial magnetosomes also highly attractive as biomaterial in several biotechnical and biomedical applications, such as magnetic imaging [4] and hyperthermia [5–7], as well as magnetic separation and drug targeting [8–10].

In the well-studied alphaproteobacterium *Magnetospirillum gryphiswaldense* and related MTB, magnetosomes consist of a monocrystalline core of magnetite (Fe_3_O_4_) bounded by a dedicated proteo-lipid membrane [2, 11]. Magnetosome biosynthesis starts with the invagination of the magnetosome membrane (MM) vesicle, followed by sorting of specific magnetosome proteins into the MM, the accumulation of large amounts of iron within the MM vesicles and the biomineralization of well-ordered crystals of magnetite (Fe_3_O_4_), and finally, their assembly and positioning into linear chains along the dedicated cytoskeletal network [2, 12].

Magnetosome biosynthesis has been found to be orchestrated by numerous proteins [2], which together build a sophisticated machinery that exerts strict control over each step of magnetosome formation. Most specific functions are encoded by the > 30 genes termed *mam* (magnetosome membrane), *mms* (magnetosome particle membrane-specific) and *feoAB1* (a magnetosome-specific Fe^2+^ transport system) [2, 13, 14]. These are all clustered in five major operons within a larger genomic magnetosome island (MAI) that extends over ~ 110 kb [14, 15]. Transfer of all five *mam*- and *mms*-operons (MagOPs) from *M. gryphiswaldense*, conferred magnetosome biosynthesis to various foreign, hitherto non-magnetic bacteria, thereby confirming the essential role of this gene set [16, 17]. A recent analysis by RNA-sequencing, bioluminescence reporter assays and promoter knockouts revealed that the transcriptional architecture of magnetosome operons is complex [18]: in microaerobically grown cells, the *mamGFDCop* (2.1 kb) and *feoAB1op* (2.4 kb) operons are transcribed as single transcriptional units, whereas multiple transcription start sites (TSS) were present in the *mms6op* (3.6 kb), *mamXYop* (5 kb) and the long *mamABop* (> 16 kb), which comprises 17 genes and encodes all the essential factors for magnetosome biosynthesis [13, 19].

An increasing number of studies indicated that in addition to key functions encoded by the MAI genes, further auxiliary genes encoding generic cellular functions located outside the MAI are required for proper magnetosome biosynthesis. For example, aerobic and anaerobic respiration pathways were shown to participate in magnetite biomineralization, probably by contributing to oxidation of ferrous iron to ferric iron under oxygen-limited conditions [20, 21]. Mutants of *M. gryphiswaldense* that lack enzymes of the denitrification pathway, such as the periplasmic nitrate reductase (NapAB), Fe^2+^–nitrite oxidoreductase (NirS) or nitric oxide reductase (NorBC) were severely impaired in magnetite biomineralization [20, 21]. The importance of respiratory pathways was confirmed by a genome-wide transposon mutagenesis screen, in which also further genes with additional auxiliary functions were implicated in magnetosome biosynthesis, such as sulfate assimilation, oxidative protein folding and cytochrome c maturation [22].

In addition to the availability of micromolar amounts of iron [23, 24], the O_2_ concentration was found to be the crucial factor affecting magnetite biomineralization in *M. gryphiswaldense* [25, 26]. Magnetite crystals are formed only under microoxic to anoxic conditions, whereas dissolved oxygen concentration (dO_2_) > 10% air saturation were found to entirely inhibit the formation of magnetosomes [25, 26]. However, the molecular mechanisms and determinants of oxygen regulation and redox control of magnetite biomineralization have remained unclear. Several early studies suggested that the transcription of magnetosome genes comprised in the MagOPs is only weakly affected by oxygen (and iron) [13, 27]. This was also observed in a whole-transcriptomic study by Wang and colleagues, which found that MagOP expression was neither affected by oxygen nor iron. Whereas genes coding for iron regulation, transport and metabolism were differentially expressed under high iron conditions, oxygen mainly affected genes encoding nitrate respiration [28, 29].

However, although these previous studies already revealed valuable insights into the transcriptional organization and the role of oxygen, major parts of the regulation and transcriptional architecture are still unknown. Most importantly, previous studies [18, 28] employed microoxic conditions supporting fastest growth, but suboptimal magnetosome formation, as indicated by the formation of fewer, less regular and smaller magnetosomes compared to anoxic conditions [25, 26]. Furthermore, the operon architecture and transcriptional organization of genes involved in magnetosome biosynthesis outside the MAI has remained unknown. In addition, given the importance of *M. gryphiswaldense* as a widely studied model organism for biomineralization, organelle formation and magnetotaxis, knowledge about global regulatory features, such as promoter and operon structures within the MAI and in the entire genome is needed.

Here, we studied the transcriptional profiles of *M. gryphiswaldense* during magnetosome biomineralization under anaerobic conditions, favoring highest magnetite biomineralization, compared to oxic conditions entirely inhibiting magnetite synthesis [25, 26]. In addition, we present new candidates for further magnetosome biosynthesis-associated genes, and reveal genome-wide structures and positions of promoters, operons and other regulatory elements.

## Results

### Cultivation and RNA-sequencing of *M. gryphiswaldense*

**Fig. 1:**
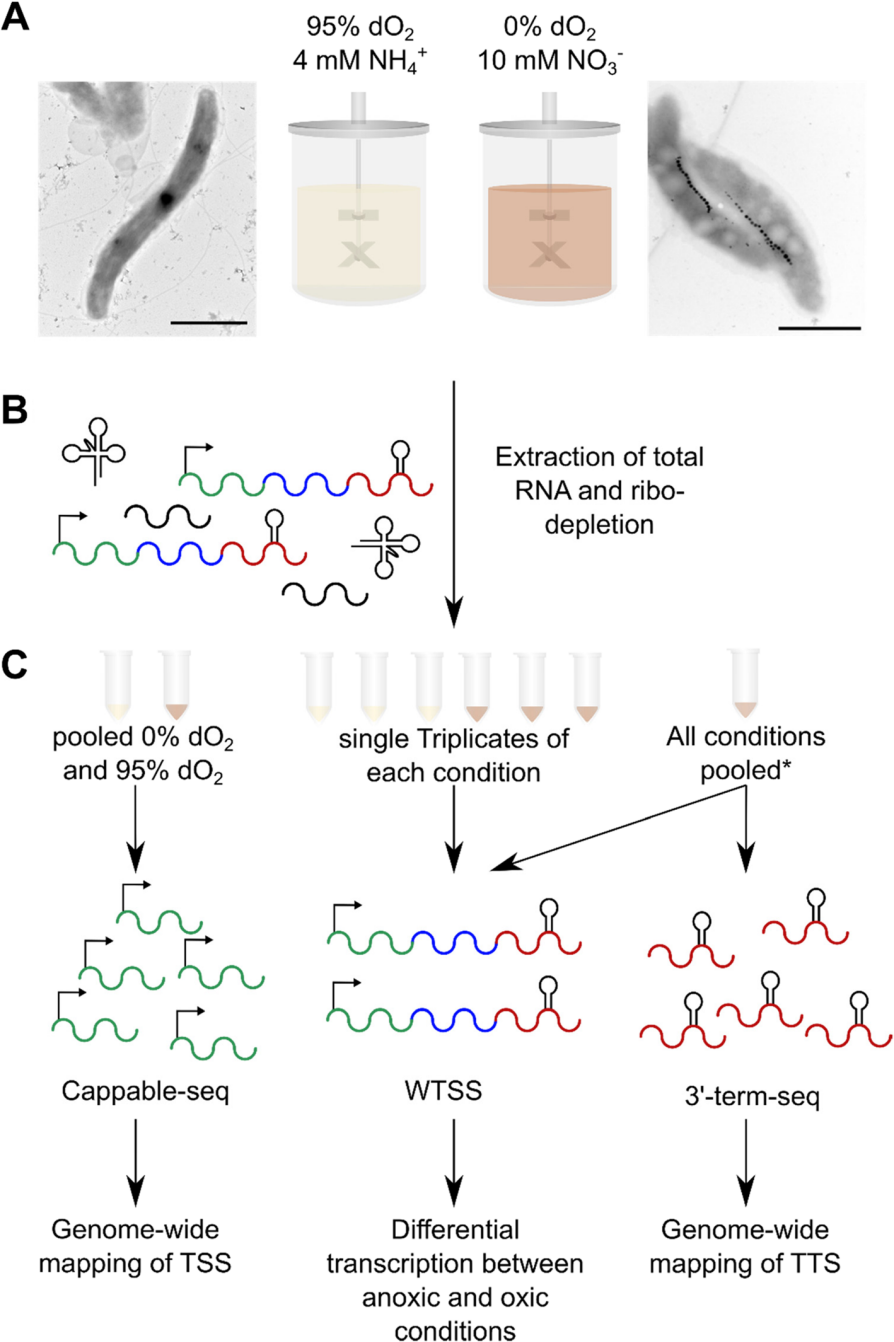
Overview of the study design. **A** Cultivation of *M. gryphiswaldense* under controlled oxic (95% dO_2_) and anoxic (0% dO_2_) conditions resulting in nonmagnetic and magnetosome forming cells, respectively, as visible by transmission electron microscopy imaging (scale bar 1 μm). **B** Extraction of the total RNA in technical triplicates, followed by **C** pooling of the samples for the three different library preparations andlibrary preparation prior to RNA-sequencing. * All conditions encompassing anoxic, microoxic and oxic growth with nitrate (NO_3_^−^) or ammonium (NH_4_^+^) were pooled for this analysis

To compare transcriptomic profiles between magnetic and non-magnetic cells, we mainly focused on the analysis of cells grown under two conditions: i) the entire absence of oxygen (0% dO_2_) with 10 mM nitrate as electron-acceptor for anaerobic denitrifying growth [20]. These anoxic conditions are known to support optimal magnetosome biosynthesis [20, 25, 26]. For comparison, cells were grown under ii) oxic conditions (95% dO_2_), which were shown to entirely suppress magnetosome biosynthesis [24, 25]. In addition, the anaerobic electron acceptor nitrate was replaced by an equimolar amount of ammonium as the nitrogen source (see Fig. 1 for a summary of growth experiments and library construction). For each condition, cells were cultured in triplicates at 28 °C within an oxystat fermenter allowing precise control of all growth parameters [26]. Anoxic cultures reached a final optical density (OD_565_) of approximately 0.5 after 25 h (Fig. S1), and as expected, exhibited the highest magnetic response (*C*_mag_, a light-scattering proxy for magnetosome biomineralization [30]) of > 0.7 as well as the largest average crystal size (34 nm) with 25 magnetosomes per cell (Fig. 1). For comparison, oxic cultures grew to an OD_565_ of 0.8 after 26 h and did not form magnetite crystals, as indicated by a *C*_mag_ of nearly 0 and the absence of electron dense particles in electron micrographs in most cells (Fig. 1). Growth was highly consistent between all replicates per condition (Fig. S1).

To minimize putative effects of media depletion, we chose sampling points during early growth, which was OD_565_ 0.1 for anoxic, and OD_565_ 0.2 for oxic conditions. Upon sampling, triplicates from anoxic and oxic conditions were pooled, respectively, and used for genome-wide TSS identification by Cappable-sequencing [31]. In addition, for the genome-wide identification of transcription termination sites (TTSs) as well as elucidation of operon structures, 3’end-sequencing technique [32] and whole transcriptome shotgun sequencing (WTSS) were applied. The WTSS libraries were separately constructed from each replicate of conditions i) 0% dO_2_ with 10 mM nitrate and ii) 95% dO_2_ for evaluation of differential transcription. For the detection of maximal numbers of operons and termination events, results from two additional conditions were considered: iii) microoxic conditions (1% dO_2_, 4 mM nitrate) as used for high yield routine cultivation and magnetosome production [25], iv) as well as oxic conditions (95% dO_2_) with 4 mM nitrate as an alternative nitrogen source to separate effects of electron acceptor from nitrogen-source (Fig. S1). Samples of triplicates from all four growth conditions were pooled and used for WTSS and 3′ end-sequencing.

### Effects of anoxic growth conditions permitting magnetosome biosynthesis on gene expression

**Fig. 2:**
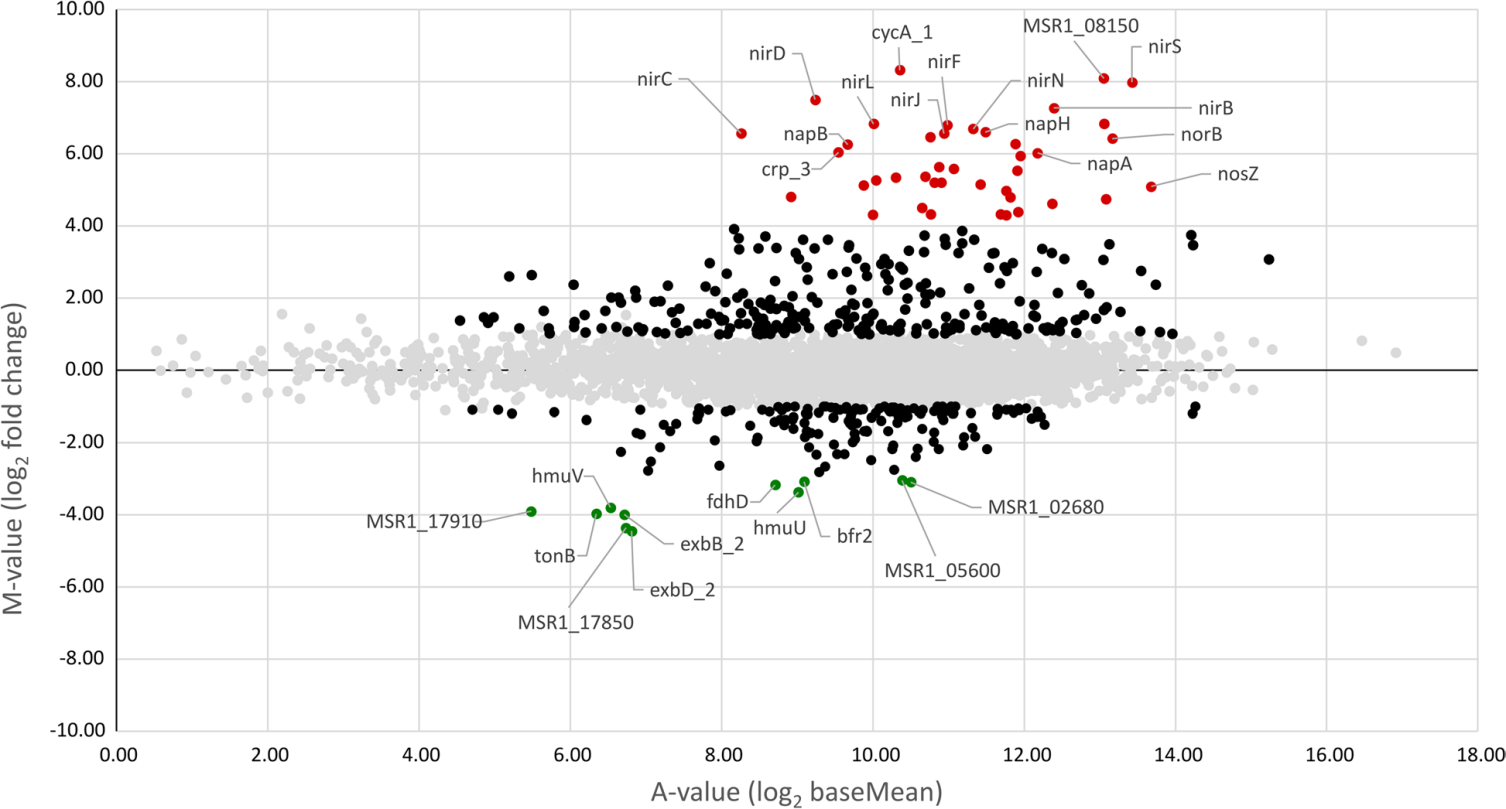
Differential expression of genes under anoxic (0% dO_2_) vs. oxic (95% dO_2_) conditions with upregulated and downregulated (M-value ≤ − 1, M-value ≥1, black dots), highly upregulated (M-value ≥4, red dots), and highly downregulated (M-value ≤ − 3, green dots) genes under anoxic conditions. The grey dots represent unsignificant differential expression (M-value ≤1, M-value ≥ − 1)

We first compared the genome-wide abundance of transcripts under anoxic and oxic conditions. For the identification of highly differentially expressed genes, the M-value (log_2_ of the calculated foldchange) was plotted against the A-value (log_2_ of the base mean) as proxy for the expression level of each gene (Fig. 2). From the > 4300 genes in total, about 300 were found significantly upregulated.

To capture only the most significantly regulated genes, the M-value threshold of ≥4 for stronger and ≤ − 3 for downregulated genes were qualitatively chosen from the MA-plot (Fig. 2). Using these thresholds, 41 genes were found highly upregulated in magnetic cells under anoxic conditions and 11 genes downregulated compared to oxic conditions (Table S3).

Highest upregulation of all (319.6 -fold, M-value: 8.32) was detected for *cycA_1*, which encodes a cytochrome c4-precursor. Further genes highly upregulated (16 to 256-fold, M-value 4–8, see Table S3 for details) in anoxic magnetic cells are linked to various steps of denitrification such as *napABCHG* (nitrate reduction) *nirCDEFGHJLST*, *nnrS* [33] (nitrite reduction), *norBCDQ* (nitric oxide reduction) and *nosZ* (nitrous oxide reduction) [20, 21, 34].

Expression of various oxygen-dependent cytochrome c terminal oxidases was also affected by anoxic conditions: genes *ccoNOQP* encoding the subunits of the *cbb3*-type cytochrome c oxidase [35], were upregulated under anoxic conditions by 2.1, 2.4, 2.2 and 1.6-fold (M-value 1.04, 1.27, 1.12 and 0.66), respectively. In previous studies, an *aa3*-type cytochrome c terminal oxidase was found only active under oxic conditions [35]. Consistently, we found *coxBAC* and *ctaG* encoding this oxidase downregulated by 4.2, 6.7, 5.0 and 6.2-fold (M-value − 2.07, − 2.75, − 2.32 and − 2.63), respectively. A third type of cytochrome oxidase, the *bd*-type cytochrome c oxidase, encoded by the genes *cydBA* [35], was actively transcribed under anoxic conditions with an A-value of ca. 7 but genes were not differentially expressed across conditions. Further highly upregulated genes were MSR1_08970 (M-value 5.27) and MSR1_08150 (M-value 8.09), which may putatively encode a cytochrome c and a cytochrome c oxidase, supported by their co-localization with genes of related functions within common operons. However, no heme-binding motif (CXXCH) was found during sequence analysis.

Some of these respiratory genes were previously found to be regulated by the oxygen-sensing transcription factor called MgFnr, which represses denitrification genes with increasing oxygen concentration [36]. We found *Mgfnr* itself to be only weakly regulated (M-value − 0.44), thus ensuring the presence of this regulator under all growth conditions. Two other homologues of the *fnr*-family MSR1_08370 and MSR1_08380 were highly upregulated in magnetic cells (M-value 6.04 and 2.37), thus representing additional potential regulators of magnetosome biosynthesis-associated genes.

Because of the involvement of many cytochrome c-like proteins in magnetosome biosynthesis [2, 31, 35, 37, 38], and also their abundance among highly upregulated genes in anoxic magnetic cells, we further focused on genes responsible for cytochrome c biosynthesis and maturation. For example, *resA,* which enables heme to bind by breakage of the disulfide bonds in apocytochrome c [39], was highly upregulated by 19.8-fold (M-value 4.31). The genes *ccmG* (disulfide bond formation) and *ccmI* (apo-cytochrome c chaperon) [40] from the operons *ccmABop* and *ccmCDEFGHIop* were weakly, but significantly downregulated by 1.4 and 1.2-fold (M-value − 0.51 and − 0.26), respectively, whereas *ccmA* was 1.4-fold upregulated (M-value 0.44). Transcription of other genes from these operons remained unchanged between anoxic and oxic conditions. Further genes that are associated with cytochrome c biogenesis and were previously implicated in magnetosome biosynthesis [22] are *dsbA* and *dsbB*, which function in disulfide bond formation during translocation of proteins across the cytoplasmic membrane [41]. However, their transcription was essentially unaffected between anoxic and oxic conditions.

Another highly upregulated gene (49.9-fold, M-value 5.64) was MSR1_19280, which encodes an *HHE cation binding domain* containing protein with unknown function. This domain is found in bacteriohemerythrins known for binding oxygen during import processes, but is also found in proteins that play a part in transcriptional regulation in response to oxygen or nitrate [42, 43]. Several other hemerythrin-like genes were upregulated under anoxic conditions, including MSR1_34750, MSR1_33560 and MSR1_04470 by 4.0, 2.3 and 18.7-fold (M-value 2.00, 1.22 and 4.32), respectively.

Among the most highly downregulated genes under anoxic conditions were *exbD_2* (M-value − 4.45), *exbB_2* (M- value − 3.99) and *tonB* (M-value − 3.97), all of them involved in import of various substrates, including iron siderophores [44]. Likewise, *hmuV* and *hmuU* involved in the import of hemin, another putative iron source, were also downregulated 13.9 and 10.3-fold (M-value − 3.80 and − 3.37), respectively. Since ferrous iron becomes oxidized to insoluble ferric iron in the presence of oxygen, this might lead to the exploitation of alternative iron sources (e.g. siderophores and heme) in anticipation of iron shortage under oxic conditions. On the other hand, the lower transcription of siderophore and heme uptake genes under anoxic conditions suggests only a minor role of these proteins in magnetosome biosynthesis. Other genes with a function in iron homeostasis are bacterioferritins *bfr1* and *bfr2*, which were previously implicated in magnetosome biosynthesis by Mößbauer spectroscopy [45]; this, however, was questioned more recently by a genetic approach [46]. Here, single and double deletions of *bfr1* and *bfr2* did not impact magnetite formation in *M. gryphiswaldense* [46]. Consistent with the latter *bfr1* and *bfr2* were downregulated under anoxic conditions by − 7.0 and − 8.5-fold (M-value − 2.81 and –3.08), respectively. However, this interesting observation does not necessarily indicate whether bacterioferritins are involved in magnetosome biosynthesis or not. Since the oxic conditions were likely to impose oxidative stress to the microaerophilic *M. gryphiswaldense*, we expected genes involved in tolerance to reactive oxygen species to be among the differentially transcribed genes (Fig. 2). In fact, *tpx* and *sodB*,coding for putative peroxidases were downregulated under anoxic conditions by 3.2 and 2.3-fold (M-value − 1.68 and − 1.17), respectively. Additionally, MSR1_07950 coding for rubrerythrin, and *tsA*, a putative peroxidase, were both downregulated by 2.1 and 2.9 -fold (M-value − 1.04 and − 1.52). Furthermore, *rpoE* (σ^24^) a sigma factor for cell envelope and oxidative stress [47, 48] was downregulated by 2.1-fold (M-value − 1.10), whereas the putative peroxide sensing transcription factor encoded by *perR_1* was significantly upregulated by 2.4-fold (M-value 1.26).

### Expression of magnetosome gene clusters

**Fig. 3:**
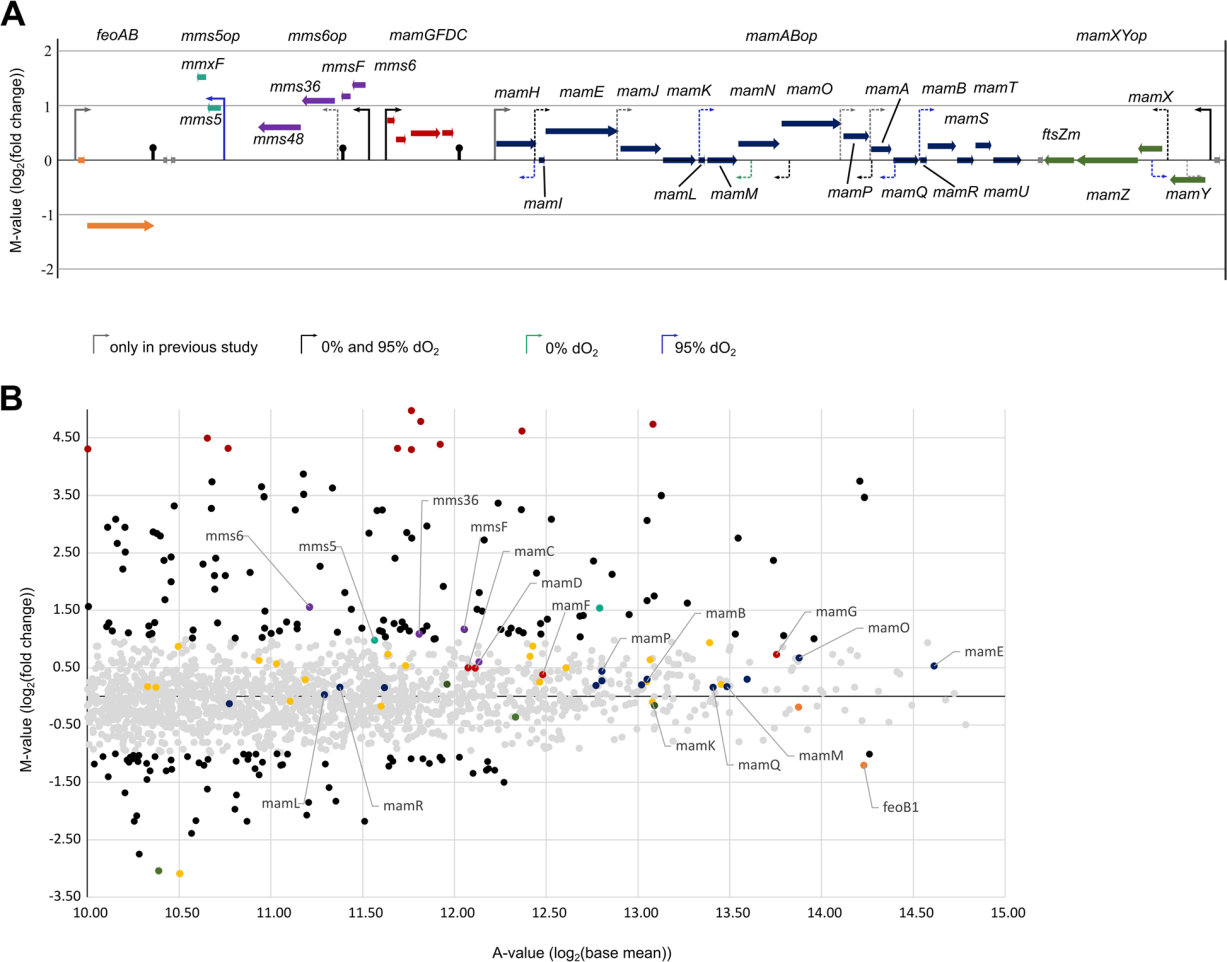
**A **Overview of the MagOPs with previously identified [18] and novel (this study) TSS. **B **Differential expression of genes under anoxic (0% dO_2_) vs. oxic (95% dO_2_) conditions with upregulated and downregulated (M-value ≤ − 1, M-value ≥1, black dots) as well as top highly upregulated (M-value ≥4, red dots) and highly downregulated (M-value ≤ − 3, green dot) genes. Grey dots indicate insignificant differential expression (M-value ≤1, M-value ≥ − 1). MAI genes are highlighted in yellow. The magnetosome gene clusters are colored as following: *feoABop* in orange, *mms5op* in light green, *mms6op* in purple, *mamGFDCop* in red, *mamABop* in blue and *mamXYop* in dark green

The well-established *mam*- and *mms*-gene clusters, which are directly linked to magnetosome biomineralization, were not among the most differentially transcribed genes by applying routinely used thresholds (i.e. M-value ≥1 or ≤ − 1). However, as indicated by their high A-value, all *mam*- and *mms*-genes were among genes with highest overall expression levels (A-value of 12–14) across all investigated conditions (Fig. 3). Transcription of *mamABop*, which harbors all essential genes for magnetosome biosynthesis [19], was largely unaffected between anoxic and oxic conditions. Strongest upregulation among *mamABop* genes was observed for *mamE*, *mamO* and *mamP* 1.4 (M-value 0.53), 1.6 (M-value 0.67) and 1.4-fold (M-value 0.44), respectively, while *mamH*, *mamJ*, *mamN*, *mamA*, *mamB* and *mamT* showed only low upregulation by 1.2-fold (M-value 0.26–0.3). Only genes from two magnetosome operons were upregulated under anoxia: 1) *mms5* and *mmxF* from the *mms5op* (M-value 1.54 and 0.98) and 2) *mms6*, *mmsF*, *mms36,* and *mms48* from *mms6op* (M-value 1.56, 1.17, 1.09, 0.6) that are all important for magnetite particle size regulation [19, 49–51]. The accessory genes *mamGFDC* [52] were only weakly upregulated ca. 1.4-fold (M-value 0.73, 0.38, 0.49, 0.5).

From *mamXYop* genes, no significant differential expression was observed for *mamZ* and *ftsZm*, while *mamY* and *mamX* showed weak, but opposite regulation patterns. Under anoxic conditions *mamY* was 1.3-fold (M-value − 0.36) downregulated, whereas *mamX* was upregulated 1.2-fold (M-value 0.21). This seems to be in agreement with their suggested functions, where a possible link between denitrification, the cellular redox potential and biomineralization was suggested for *mamX*, *mamZ* and *ftsZm* [53, 54], while *mamY* was shown to encode a cytoskeletal protein involved in magnetosome chain positioning rather than biomineralization [55]. In addition to the already observed primary promoter upstream of *mamY* (P*mamY*), an intergenic promoter (P*mamX*) between *mamY* and *mamX* was detected under both conditions, which might drive the different transcription of *mamX*, *mamZ* and *ftsZm* [18].

The *feoAB1op*, one of the two ferrous iron uptake systems present in *M. gryphiswaldense,* is thought to be mainly responsible for ferrous iron uptake for magnetosome biosynthesis [2, 56]. Under anoxic conditions, *feoB1*, which encodes a ferrous iron transporting transmembrane GTPase, was 2.3-fold downregulated (M-value − 1.2), while *feoA1* encoding a Fe^2+^ transport related protein of unknown function [57] remained unchanged.

### Genome-wide analysis of promoter architectures

Next, all TSS present under anoxic and oxic conditions were identified with Cappable-seq [31]. After empirical testing, the thresholds providing high specificity as well as reasonable reduction of false positives were set to an enrichment factor of 2.5. By applying this threshold, 5200 and 5002 TSS were identified for oxic and anoxic conditions, respectively, with 2755 (95% dO_2_) and 2579 (0% dO_2_) TSS exclusively found in each respective condition. Identified TSS were classified as primary TSS upstream of the corresponding coding regions (pTSS), intragenic TSS (iTSS), anti-sense TSS (asTSS) and other TSS (oTSS) not part of any of the classes mentioned before.

**Fig. 4:**
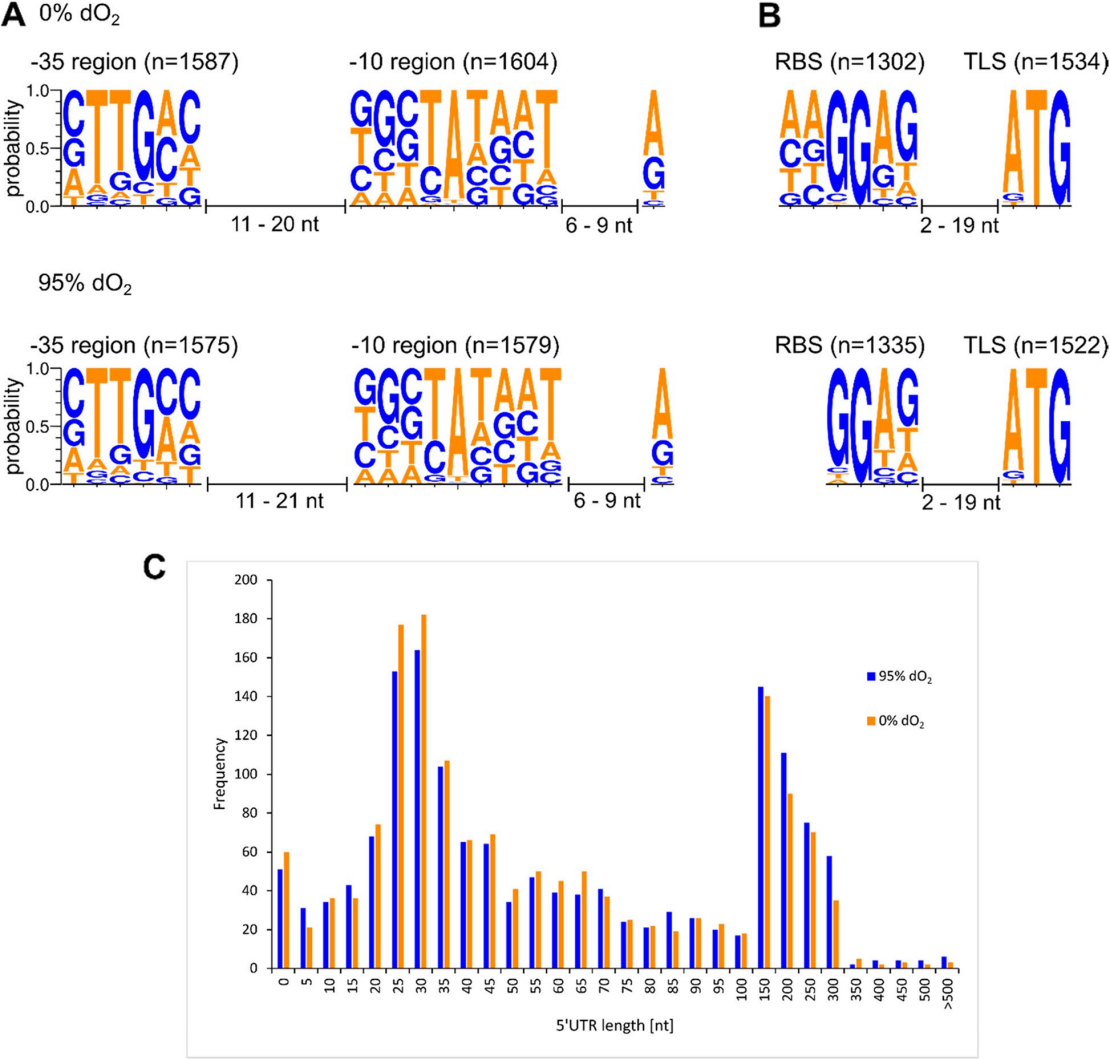
**A** Promoter architecture of identified primary transcription start sites (pTSS) under anoxic (0% dO_2_) and oxic (95% dO_2_) conditions including −35, −10 regions and TSS as well as spacer region lengths. **B** Consensus sequences of the ribosome binding site (RBS) and the translation start site (TLS) with the distances of the interspacing region. **C** Distribution of 5′ untranslated region (5’UTR) lengths for anoxic and oxic conditions. The motif logos were created with Weblogo [59]

Since promoter motifs are most highly preserved in intergenic regions, which do not obey the evolutionary restrictions of protein coding regions, motif analysis was performed with the identified pTSS using the software Improbizer [58]. Under both oxic and anoxic conditions, a conserved TATaaT motif was identified (Fig. 4A). Furthermore, a second motif was recognized with the consensus sequence of cTTGcc. Both motifs are separated by a 11–20 bp interspacing region. In most genes, transcription starts with a conserved adenine 6–9 bp downstream of the corresponding − 10 region. For both conditions, a conserved aaGGAG motif as ribosome binding site (RBS) with a 2–19 nt spacer to the start codon was detected (Fig. 4B). Consensus sequences were calculated separately for pTSS within the MAI (inMAI) and the rest of the genome (exMAI). However, no differences were found (Fig. S3).

To further identify putative regulatory elements of translation apart from the RBS, the region between the identified pTSS and the translation start site (TLS), the so called 5′-untranslated region (5′-UTR) was extracted and further analyzed (Fig. S2). From the 1522 (95% dO_2_) and 1534 (0% dO_2_) extracted 5′-UTRs, 5% (Number of 5′-UTRs under 95% dO_2_ 51 and 0% dO_2_ 60) were considered as leaderless transcripts (5′-UTR length 0–9 nt), since 5’UTRs below 9 nt are considered too short to harbor an RBS with a corresponding spacer region. Short 5′-UTRs with a length of 25–35 nt were the dominant fraction with 20.8% (317) and 23.4% (353) of the investigated sequences under oxic and anoxic conditions, respectively (Fig. 4C). These 5′-UTRs are sufficiently long to comprise an RBS with the corresponding spacer to the TLS.

A second dominant fraction (406 5′-UTRs under 95% dO_2_ (26.7%), and 353 under 0% dO_2_ (23.0%)) ranging from 150 to 300 nt in 5′-UTR length was identified. This suggests a high degree of regulation at both the transcriptional and translational level by cis-regulatory elements such as riboswitches, secondary structures or attenuators since 5′-UTR of these lengths are known to enable such complex structures [60, 61]. Analysis of the genome sequence with the Rfam database [62, 63] identified three putative riboswitches (Table 1), in addition to the previously identified putative regulatory elements in the 5′-UTR [15]. Additionally, one small RNA (sRNA) was identified in the genome.

**Table 1:** Predicted cis-regulatory elements by using the Rfam database [62, 63]

Name	Start	Stop	Bit Score	Strand	0% dO_**2**_ TSS	95% dO_**2**_ TSS
Cobalamin riboswitch	530,889	531,128	118.2	+	530,905	530,801
Guanidine-I riboswitch*	119,571	119,680	75.6	–		119,678
SAM riboswitch	3,457,791	3,457,868	69.5	–	3,457,976	3,457,976
Glycine riboswitch	3,661,824	3,661,919	68.7	+	3,661,819	3,661,819
TPP riboswitch	72,546	72,658	70.6	+	72,467	72,558
manganese riboswitch*	2,189,415	2,189,528	49	–	2,189,523	2,189,523
rpsB*	1,038,575	1,038,671	46.8	+	1,038,568	1,038,569
Guanidine-II riboswitch*	3,082,026	3,082,072	45.7	+	3,082,019	3,082,019
BjrC80 sRNA*	1,456,741	1,456,921	43.4	–		

The newly predicted cis-regulatory elements are highlighted by an asterisk

### Elucidation of the global operon architecture

**Fig. 5:**
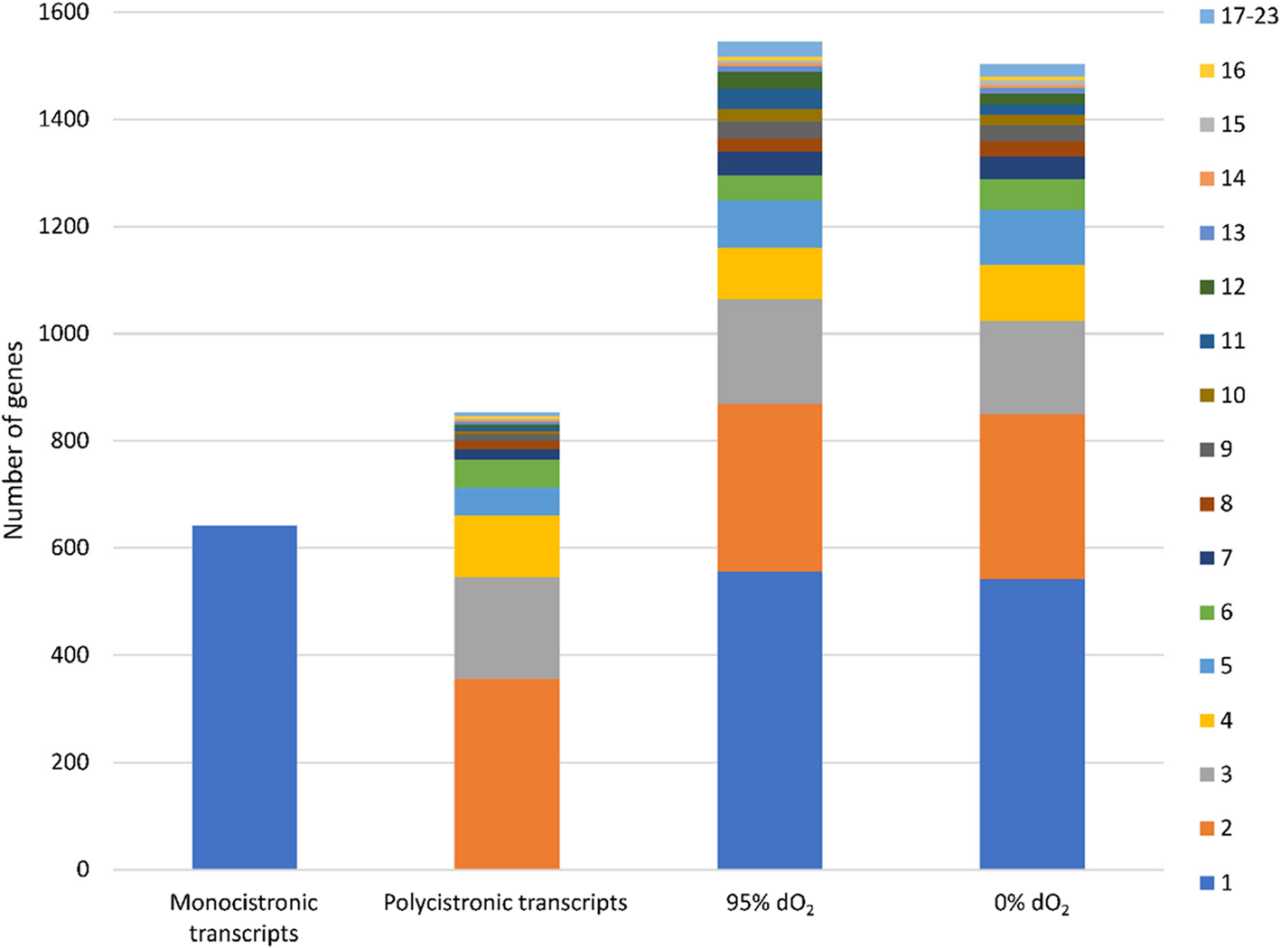
Distribution of identified operons by gene number. Monocistronic and polycistronic transcripts were identified from the pooled dataset including all tested conditions. Sub-operons were defined by transcription start site (TSS) identification within a primary operon for the respective condition (95 and 0% dO_2_). The number of genes within the monocistronic and polycistronic as well as the sub-operons is coded by color

We further investigated genome-wide operon organization by combining Cappable-seq, WTSS as well as 3′-end-sequencing. To enhance the detection of operons and termination events, results from microoxic and oxic conditions, with nitrate as a nitrogen source, were considered, in addition to the main oxic and anoxic conditions. Initial analysis was conducted using the automatic operon prediction tool as part of the ReadXplorer software [64] with the threshold of at least three spanning reads for assigning two neighboring genes into a primary operon (i.e., a polycistronic transcript harboring at least two genes). If additional TSS were located within a presumed primary operon, a sub-operon was assumed. Using this procedure, 643 genes were found expressed as monocistronic transcripts, and 853 as primary operons comprising between 2 and 23 genes, and 3254 genes in total (Fig. 5). The majority (89% / 764 primary operons) of the polycistronic transcripts comprised 2–6 genes with 357 primary operons coding for two genes (41.9%), followed by transcripts with three genes (22% / 189 primary operons). Among the longest operons was the well-studied *mamABop* with 17 genes involved in magnetosome biosynthesis. Other examples for long operons comprised 16 genes (17 kb) encoding NADH-quinone oxidoreductase subunits, and 21 genes encoding ribosomal proteins (9 kb).

Different sub-operon profiles were identified in magnetic (anoxic) and non-magnetic (oxic) cells. Under oxic conditions 1545 sub-operons were identified with 814 sub-operons exclusive for this condition. Anoxic datasets showed 1504 sub-operons of which 836 were exclusively identified under this condition. In both conditions, the majority of sub-operons encompass a single gene (oxic: 36.1% /anoxic: 36.1%), followed by two genes (20.1% / 20.4%), whereas 43.8% / 43.8% of the identified sub-operons consist of more than three genes (Fig. 3).

Within the MAI, 28 monocistronic transcripts and 30 primary operons (including the MagOPs) were identified under all four investigated conditions, the majority of which encompass two genes (21 primary operons). Furthermore, 47 sub-operons dataset were identified within the oxic dataset, whereas only 29 within the anoxic dataset, suggesting a lower transcriptional complexity under anoxic conditions. Whereas the architecture of the two primary *feoAB1op* and *mms5op* did not change, showing two and no sub-operons, respectively, the other MagOPs exhibited condition-dependent changes in TSS numbers resulting in different sub-operonic structures. The smaller primary operons *mms6op* (3 / 2) and *mamGFDCop* (2 / 1) showed a decreased complexity by one sub-operon under anoxic conditions. The most striking effect was observed for *mamABop*, in which the number of 36 sub-operons under oxic conditions decreased to only 13 under anoxic conditions. The same was observed in the case of *mamXYop*, where the number of sub-operons decreased from 6 to 3 sub-operons.

## Discussion

Here, we employed a combination of various RNA-seq techniques to identify the transcriptional landscape, promoter structure and operon architecture of *M. gryphiswaldense* during anaerobic conditions favoring magnetosome formation. As expected, many of the upregulated genes are directly or indirectly linked to anaerobic respiration and most of these genes (23) have functions in various steps of denitrification. Determinants of aerobic respiration were also among the top upregulated genes in anoxic magnetic cells, such as the *cbb3* oxidase encoded by *ccoNOQP* operon. Besides encoding the primary cytochrome c terminal oxidase for aerobic respiration, *cbb3*-type oxidase was also linked to the redox balance control required for magnetosome biomineralization, which was severely impaired upon deletion [35]. By contrast, the *aa*_*3*_-type cytochrome c oxidase encoded by *coxBAC* and *ctaG* with a suggested function in oxygen detoxification, but no role in magnetosome biosynthesis [35] was significantly downregulated, and the *bd*-type cytochrome c terminal oxidase encoded by *cydBA* did not show any differential expression. Additionally, we also found several genes involved in cytochrome c maturation and disulfide bond formation among upregulated genes. Since several key proteins involved in magnetosome biosynthesis are c-type cytochromes exhibiting a unique so called “magnetochrome” fold [37, 38], regulation of the cytochrome c maturation system may affect magnetosome biosynthesis directly, in addition to the more indirect effects on many cytochrome c domain containing respiratory enzymes. Indeed, genetic impairment of cytochrome maturation resulted in aberrant magnetite crystal morphologies in a genome wide transposon mutagenesis screen [22]. The genes *dsbA* and *dsbB* are involved in the proper folding of periplasmic proteins through disulfide bond formation [65], but also have a suspected auxiliary function in magnetosomes biosynthesis [22]. For example, several magnetosome proteins, such as MamE/F/G/H/N/P/S/T/X/Z, contain more than two cysteines in their proposed luminal domains [66], rendering them putative substrates of DsbA and DsbB. In our analysis, the constitutive high expression of *dsbA* and *dsbB* (A-value 9–10) would agree with such an important function.

Genes for respiratory functions were also found enriched among the 77 upregulated genes in microoxic (0.5% dO_2_) magnetic *M. gryphiswaldense* cells compared to semi-oxically grown cells (30% dO_2_) by Wang and colleagues [28]. In the same study, 95 genes, involved in various generic cellular processes, were downregulated under 0.5% dO_2_ compared to cells grown under 30% dO_2_ [28]. The top upregulated *cycA_1* and putatively transcriptional regulators such as *crp_1* or *crp_3* identified in our study escaped detection in the previous study [28]. Furthermore, motility associated genes found to be highly regulated by Wang et al. [28], were not among the top-upregulated genes in our study. Since overlapping expression of aerobic and anaerobic key genes was observed under microoxic conditions [20], our comparison between highly controlled inhibitory oxic and fully anoxic conditions favoring highest magnetite biomineralization can be expected to reveal more pronounced regulatory differences between magnetic and non-magnetic cells, which likely explains the higher number of differentially expressed genes (300 up-, and 164 downregulated) identified in our study. Among them, several genes with so far unknown function, such as MSR1_19280, MSR1_19290 and MSR1_04470 represent novel candidates for respiration-linked genes, but as such might also be putatively involved in magnetosome biosynthesis because of their high upregulation in magnetic cells.

Hemerythrins were previously implicated in magnetosome biosynthesis because of their known function in oxygen sensing as well as iron transport in other bacteria [43]. In addition, the conspicuously high numbers of genes encoding bacteriohemerythrins present in the genome (26 copies), and in particular within the MAI of *M. gryphiswaldense* led to speculations about a possible function in magnetosome biosynthesis [42, 67]. Thus, their upregulation in magnetic ells observed in our and a previous study [28] would be consistent with such a function, which however needs to be further investigated.

### Regulation of magnetosome genes

A remarkable finding was that the magnetosome specific genes comprised within the *feoAB1op*, *mms5op*, *mms6op*, *mamGFDCop*, *mamABop* and *mamXYop* operons were among the most highly transcribed genes in the cell, comparable to, or even exceeding highly expressed housekeeping genes, such as those coding for DNA-polymerase subunits (*dnaE* and *dnaN*, A-value 12.2 and 12.1), as well as ribosomal proteins (*rplD* and *rplE*, A-values 13.0 and 11.4). The overall weak regulation of the majority of the *mam*- and *mms*-genes confirmed earlier studies, which suggested a constitutive expression of specific magnetosome biosynthesis genes [13, 28]. In addition, previous studies showed that key magnetosome proteins as well as empty magnetosome membrane vesicles were highly abundant in non-magnetic cells in which magnetite biomineralization was entirely suppressed by aerobic cultivation [13, 68]. Expression of the large *mamABop* encoding key functions in magnetosome membrane formation, assembly and crystal nucleation, remained largely unchanged at high expression levels between our tested conditions. Only *mms6op*, *feoAB1op* and *mms5op*, which are not essential, but have redundant or accessory function in iron transport or magnetite crystal size regulation, were upregulated in magnetic cells. The high and constitutive expression of magnetosome genes indicates that magnetosome biosynthesis is among the key cellular functions under all conditions. Thus, the absence of magnetite crystals in oxic cells cannot be explained by the lack or poor transcription of magnetosome specific proteins but instead possibly by abiotic direct oxidation of the cellular ferrous iron, thus disturbing the proper Fe^2+^/Fe^3+^ ratio required for magnetite precipitation, which cannot be compensated by the cellular reductase activities. Alternatively, or in addition, highly aerobic conditions may damage oxygen-sensitive cofactors important for the magnetite biomineralization, such as Fe-S-cluster containing respiratory enzymes, as observed by Imlay and colleagues in *E. coli* [69, 70].

### Oxic conditions cause increasing transcriptional complexity

We found substantial differences in the number and position of TSSs between anoxic and oxic conditions, and to some degree, also between each of those and a previous study [18]. A higher number of TSSs (5200) was identified in oxic, non-magnetic cells vs. 5002 in anoxic magnetic cells. A possible reason for the increased number of TSS in oxic cells could be the compensation of instability of long transcripts induced by reactive oxygen species, thus possibly ensuring transcription from additional sites within the operon. However, since we identified only a 4% difference in TSS-number between conditions with pooled replicates, it might be worth to clarify oxygen impact on transcriptional organization in future studies. Within the MAI, anoxic conditions also resulted in fewer TSSs than detected in the previous study [18], whereas under oxic conditions most of the previously detected TSSs were confirmed [18]. The absence of the previously detected intergenic TSS upstream of *mms36* [18] suggests that this TSS is only active under the rather undefined oxygen conditions used in the previous study [18], resulting in cells at diverse stages of growth.

### Comparative analysis of global promoter structure

Under both anoxic and oxic conditions, conserved motifs at − 35 (cTTGcc) and − 10 regions (TATaaT) separated by an interspacing region of 11–20 bp were detected. A similar promoter architecture was also identified in the σ^70^-dependent promoters in other Alphaproteobacteria such as *Gluconobacter oxydans* [71], and with sequence similarity to the − 35 (TTGACA) and − 10 (TATAAT) motifs characteristic for the *E. coli* house-keeping sigma factor σ^70^ [72]. Thus, the vast majority of *M. gryphiswaldense* primary promoters during the investigated growth phase conditions is likely σ^70^-dependent as well. The identification of conserved promoter structures also has practical implications. For instance, the P*mamDC*_45_ promoter driving transcription of the *mamGFDCop* shows a canonical σ^70^ promoter architecture with TTCGC for − 35 region and TAAATT for − 10 region separated by an approximately 20 bp spacer, and a 6 bp spacer to the corresponding TSS [13]. The high similarity between P*mamDC*_45_ sequence to the promoter motifs that we found to exhibit highest activity confirms that the P*mamDC*_45_ represents an appropriate promoter for high expression in *M. gryphiswaldense* [10, 53, 73].

Within the 5′-UTR we found a conserved aaGGAG motif serving as an RBS with in average 8 nt as spacer to the corresponding start codon in both oxic and anoxic datasets. This architecture resembles the optimized RBS (AGGAG followed by an 8 nt spacer) for expression in *M. gryphiswaldense*, which has been experimentally identified [10, 73]. Noteworthy, longer 5′-UTRs were more common in transcripts from oxic conditions, whereas significantly shorter 5′-UTRs were found under anoxic conditions. This may suggest a higher potential for regulation by cis-regulatory elements and is consistent with the increased transcriptional complexity in oxic cells, possibly suggesting an overall increased regulatory potential under these conditions. Our analysis of 5′-UTR for regulatory RNA structures, based on the Rfam database, predicted new riboswitches in addition to the previously annotated ones in the most recent version of the *M. gryphiswaldense* genome [15]. New elements with a clear regulatory function, such as a glycine-riboswitch upstream of the glycine degradation system (*gcvTHPAPB*-operon), but also elements with so far unknown function were detected such as the BjrC80 sRNA upstream of hypothetical proteins. Although some new cis-regulatory elements were found, they only represent a relatively small fraction (2.5% of 5′-UTR length 150–300 nt under anoxic conditions) compared to the high number of long 5′-UTRs (26.7 [95% dO_2_]/23% [0% dO_2_]), suggesting that most regulators remain unidentified. Taken together, it seems that expression regulation under the tested conditions in *M. gryphiswaldense* is based to a significant degree on cis-regulatory elements like riboswitches as sensors for environmental cues, as suggested by the 5′-UTR length.

## Conclusions

The transcriptome under conditions of highest magnetosome biosynthesis revealed an interplay between generic metabolic processes, such as anaerobic respiration, as well as increased biosynthesis and maturation of cytochrome c proteins and hemerythrins; some of these pathways have already been implicated in magnetosome biosynthesis. In addition, in highly magnetic cells, the transcriptional complexity is reduced compared to oxic, nonmagnetic cells. Furthermore, magnetosome genes mostly exhibit a constitutively high expression, which is only weakly affected by growth conditions.

Our study sheds light on the genome-wide complex transcriptional organization during magnetosome biosynthesis as a model for the formation of an intricate prokaryotic organelle with relevance for the research at system level. Furthermore, the insights can be used for engineering promoters as well as entire cellular pathways, thereby enabling rational design of synthetic magnetosome operons for targeted magnetosome production.

## Materials and methods

### Bacterial strains, culturing conditions and cell sampling

*Magnetospirillum gryphiswaldense* strain MSR-1 (DSM 6361) [74, 75] was cultivated in flask standard medium (FSM) comprising 10 mM 2-[4-(2-hydroxyethyl) piperazin-1-yl] ethanesulfonic acid (HEPES) (pH 7.0), 15 Mm potassium lactate, 4 mM NaNO_3_, 0.74 mM KH_2_PO_4_, 0.6 mM MgSO_4_ x 7H_2_O, 50 μM iron citrate, 3 g L^− 1^ soy peptone and 0.1 g L^− 1^ yeast extract.

Cells used for RNA isolation and cDNA library preparation were cultivated in a stirred-tank 3 L bioreactor (BioFlo™ 320, Eppendorf Bioprocess, Jülich, Germany) equipped with an InPro3253i (Mettler-Toledo, Columbus, USA) pH probe and an InPro6850i (Mettler-Toledo, Columbus, USA) O_2_ sensor, according to the previously established oxystat fermentation regime [26]. Briefly, the seed-train encompassed two passages in 10 mL FSM in 15 mL conical centrifugation tubes at room temperature for 40 h after inoculation from 4 °C stock cultures. Afterwards, stepwise scale-up was performed in screw-capped bottles with subsequent cultivation in 30 mL preculturing medium (FSM with 150 μM iron citrate and 1 g L^− 1^ soy peptone) at room temperature for 40 h followed by a second preculturing step with 300 mL preculturing medium at 28 °C for 16 h with slightly unscrewed lid for air exchange. For the second step, the incubation was performed at 120 rpm in an orbital shaking incubator, which were then used for inoculation of the bioreactor.

Oxystat fermentations were conducted under oxic (95% dO_2_), microoxic (1% dO_2_) and anoxic (0% dO_2_) in large-scale medium (LSM) comprising 15 mM potassium lactate, 4 mM NaNO_3_, 0.74 mM KH_2_PO_4_, 0.6 mM MgSO_4_ x 7H_2_O, 150 μM iron citrate, 3 g L^− 1^ soy peptone and 0.1 g L^− 1^ yeast extract. For anaerobic fermentations, the medium was supplemented with additional sodium nitrate to 10 mM to further prolong the main growth phase. Prior to inoculation of the microoxic and anoxic processes, oxygen was gassed out with nitrogen. During microoxic and oxic fermentations dO_2_ was controlled by automated adjustment of agitation (100–300 rpm) and airflow (0–10 SLPM) with compressed air [26]. For anoxic conditions, the medium was continuously sparged with 0.2 standard liter per minute (SLPM) nitrogen to prevent oxygen diffusion into the system and agitation was kept constant at 100 rpm.

Cells were harvested during main growth phase by pumping 400 mL of the fermentation broth through an ice cooled silicon tube for quick cooling to 4 °C. Subsequently the cells were pelleted at 8300 g and 4 °C for 10 min using a Sorvall RC-5B Plus centrifuge (Thermo Fisher Scientific, Waltham, USA) and shock-frozen with liquid nitrogen. The cell pellets were then shipped on dry ice to Vertis Biotechnologie AG (Freising) for RNA isolation, library preparation and sequencing.

### Cell growth and magnetic response

Both optical density (OD) as measure for cell growth and magnetic response were measured with an Ultrospec2000 pro spectrophotometer at 565 nm. The magnetic response was measured according to Schüler et al., 1995 [30]. Briefly, cells were magnetically aligned perpendicular and vertical to the light beam of a photometer resulting in a change of the OD_565_. The ratio of maximal and minimal scattering intensities subtracted by 1 (*C*_*mag*_) represents the magnetic response of the cells as estimation for magnetosome biomineralization.

### RNA isolation, cDNA library preparations and sequencing

The RNA isolation, cDNA library preparation and sequencing were performed by Vertis Biotechnologie AG (Freising). Different RNA-seq techniques were employed, such as 3′-end sequencing [32], whole transcriptome shotgun sequencing (WTSS) and Cappable-sequencing [31].

For the elucidation of genome wide transcription initiation, expression coverage and transcription termination, Cappable-seq [31], whole transcriptome shotgun sequencing (WTSS) and 3′-end sequencing [32] techniques were applied, respectively.

Total RNA was isolated from samples using the mirVana RNA isolation kit (Thermo Fisher Scientific, Waltham, USA) followed by a DNase treatment step. RNA quality was checked by capillary electrophoresis.

For the identification of transcription start sites (TSS), the extracted RNA of the oxic and anoxic triplicates were pooled resulting in two pooled RNA samples for primary 5′-end enrichment by using a modified version of the Cappable-sequencing technique [31]. Briefly, 5′ triphosphorylated RNA was capped with 3′-desthiobiotin-TEG-guanosine 5′ triphosphate (DTBGTP) (New England Biolabs, Ipswitch, USA) facilitated by the vaccinia capping enzyme (New England Biolabs, Ipswitch, USA). For enrichment of the primary 5′-ends, the biotinylated RNA was applied to a streptavidin column, washed and eluted. An uncapped control was also applied to the column to check for unspecific binding to the column matrix. Subsequently, the sequencing adapter ligation, reverse transcription and PCR amplification of the cDNA were performed according to TrueSeq Stranded mRNA library instructions (Illumina, San Diego, USA).

The WTSS library preparation was performed for biological triplicates of the four investigated conditions and a pooled RNA sample of all extracted RNAs. The ribosomal RNA was then depleted by an in-house protocol (Vertis Biotechnologie AG, Freising, Germany) for the 13 RNA samples. The remaining mRNA was purified using the Agencourt AMPure XP kit (Beckman Coulter Genomics, Chaska, USA) and quality checked by capillary electrophoresis. Fragmentation of the mRNA, reverse transcription, adapter ligation and PCR amplification were performed according to TrueSeq Stranded mRNA library instructions (Illumina, San Diego, USA).

For the 3′-end library preparation a 3′ Illumina sequencing adapter was ligated to the 3′-OH ends of the rRNA depleted RNA sample prior to reverse transcription, cDNA fragmentation, sequencing adapter ligation and cDNA purification using the Agencourt AMPure XP kit (Beckman Coulter Genomics, Chaska, USA).

All cDNA libraries (in total 15 libraries) were single-end sequenced on an Illumina NextSeq 500 system (Illumina, San Diego, USA) using 1 × 75 bp read length.

### Bioinformatic methods

#### Read mapping and visualization

The sequencing reads from all 15 libraries were trimmed for sequencing adapters and low-quality bases before mapping to the current *M. gryphiswaldense* genome (Accession No. CP027526) using the CLC Bio’s Genomic Workbench software package (Qiagen, Venlo, Netherlands) with a mapping efficiency between 93 to 98% (Table S1). The resulting datasets were then visualized and investigated with ReadXplorer [64].

#### Transcriptional start site detection and motif analysis

Transcriptional start sites (TSS) were automatically detected with the Cappable-seq tools [31]. Briefly, the relative read score (RRS) is calculated for both Cappable-seq datasets by normalizing the read coverage for each base in the reference genome to the sequencing depth. Subsequently, the enrichment score for the corresponding position is calculated according to the formular enrichment score = log_2_(RRS/RRScontrol), where RRScontrol is the relative read score in the control library at the same genomic position as in the TSS enriched library. After empirical testing, the optimal threshold for highly specific TSS detection was determined with 2.5 for both datasets (oxic and anoxic conditions). Afterwards, the identified TSS were classified based on the localization in the genome by using an automated in-house script.

For identification of the conserved σ^70^-promoter motives, sequences 70 bp upstream of the assigned pTSSs were extracted and taken as input for the motif-analysis software Improbizer [58]. To identify the consensus sequence of the ribosome binding site (RBS) the region 20 bp upstream of the translation start site (TLS) assigned to a pTSS was analyzed by Improbizer.

The identified consensus sequences for the − 10 and − 35-region were visualized with WebLogo 3 [59].

#### Elucidation of operon structure

The operon detection was performed with the automated prediction tool implemented in ReadXplorer [64]. When at least three reads connecting two coding sequences were counted the corresponding genes were assigned into a primary operon. This process was continued for the following genes until no more genes could be assigned to that operon.

Sub-operons were assigned, when a TSS (pTSS or iTSS) was identified within a primary operon.

#### Differential gene expression analysis

Prior to differential expression analysis, the reads of the replicates were normalized by transcripts per kilobase million (TPM) [76] and checked by Pearson correlation coefficient (R^2^) to ensure the suitability for comparison. All replicates among each condition show R^2^-values above 0.8 indicating the high consistency among the different experiments (Table S2). Differential expression analysis was conducted with the whole transcriptome datasets cells grown under different growth conditions described under ‘Bacterial strains, culturing conditions and cell sampling’. The reads mapped to genes of three biological replicates per condition were counted by the implemented tool in the ReadXplorer software and tested for differential expression with DESeq2 [77] using default settings. In case the false discovery corrected *p*-value was below 0.01, the corresponding gene was considered as differentially expressed under the compared conditions.

## Supplementary Information

**Additional file 1: Figure S1*.*** Cell growth and magnetic response (C_mag_) under **A**) anoxic (dO_2_ 0%, 10 mM nitrate), B) oxic (dO_2_ 95%, 4 mM ammonium), **C**) microoxic (dO_2_ 1%, 4 mM nitrate), **D**) oxic with nitrate (dO_2_ 95%, 4 mM nitrate) conditions. (Scale bar 1 μm). Growth (black and grey lines) and C_mag_ (colored lines) were depicted for each replicate (circles, diamonds and triangles). The black arrow indicates the sampling timepoint for the RNA-seq experiments.

**Additional file 2: Table S3.** List of top 41 up- and downregulated genes under anoxic in comparison to oxic conditions.

**Additional file 3: Figure S3*.*** Comparison between promoter motives of TSS located within (inMAI) and outside (exMAI) of the magnetosome island, cultivated under anoxic (0% dO_2_) and oxic (95% dO_2_) conditions. The motif logos were created with Weblogo [58].

**Additional file 4: Figure S2.** Distribution of classified transcription start sites (TSS) under anoxic (0% dO_2_) and oxic (95% dO_2_) conditions in the whole genome (pTSS, primary TSS; asTSS, antisense TSS; iTSS, intragenic TSS; oTSS; other TSS).

**Additional file 5: Table S1.** Mapping statistics of the different RNA-seq datasets including the three library preparation techniques, namely Cappable-seq, whole transcriptomic shotgun sequencing and term-seq sequencing.

**Additional file 6: Table S2.** Pearson correlation coefficients R^2^ of the biological replicates.

## Data Availability

The data discussed in this publication have been deposited in NCBI’s Gene Expression Omnibus and are accessible through GEO Series accession number GSE197098, https://www.ncbi.nlm.nih.gov/geo/query/acc.cgi?acc=GSE197098.
