# Supplementary material for: The transcriptomic landscape of Magnetospirillum gryphiswaldense during magnetosome biomineralization
Source: BMC Genomics. 2022 Oct 10;23:699. doi: 10.1186/s12864-022-08913-x (PMC9549626; doi:10.1186/s12864-022-08913-x)
Supplement: Supplementary file 1 — Additional file 1: Figure S1. Cell growth and magnetic response (Cmag) under A) anoxic (dO2 0%, 10 mM nitrate), B) oxic (dO2 95%, 4 mM ammonium), C) microoxic (dO2 1%, 4 mM nitrate), D) oxic with nitrate (dO2 95%, 4 mM nitrate) conditions. (Scale bar 1 μm). Growth (black and grey lines) and Cmag (colored lines) were depicted for each replicate (circles, diamonds and triangles). The black arrow indicates the sampling timepoint for the RNA-seq experiments. [file 12864_2022_8913_MOESM1_ESM.docx]

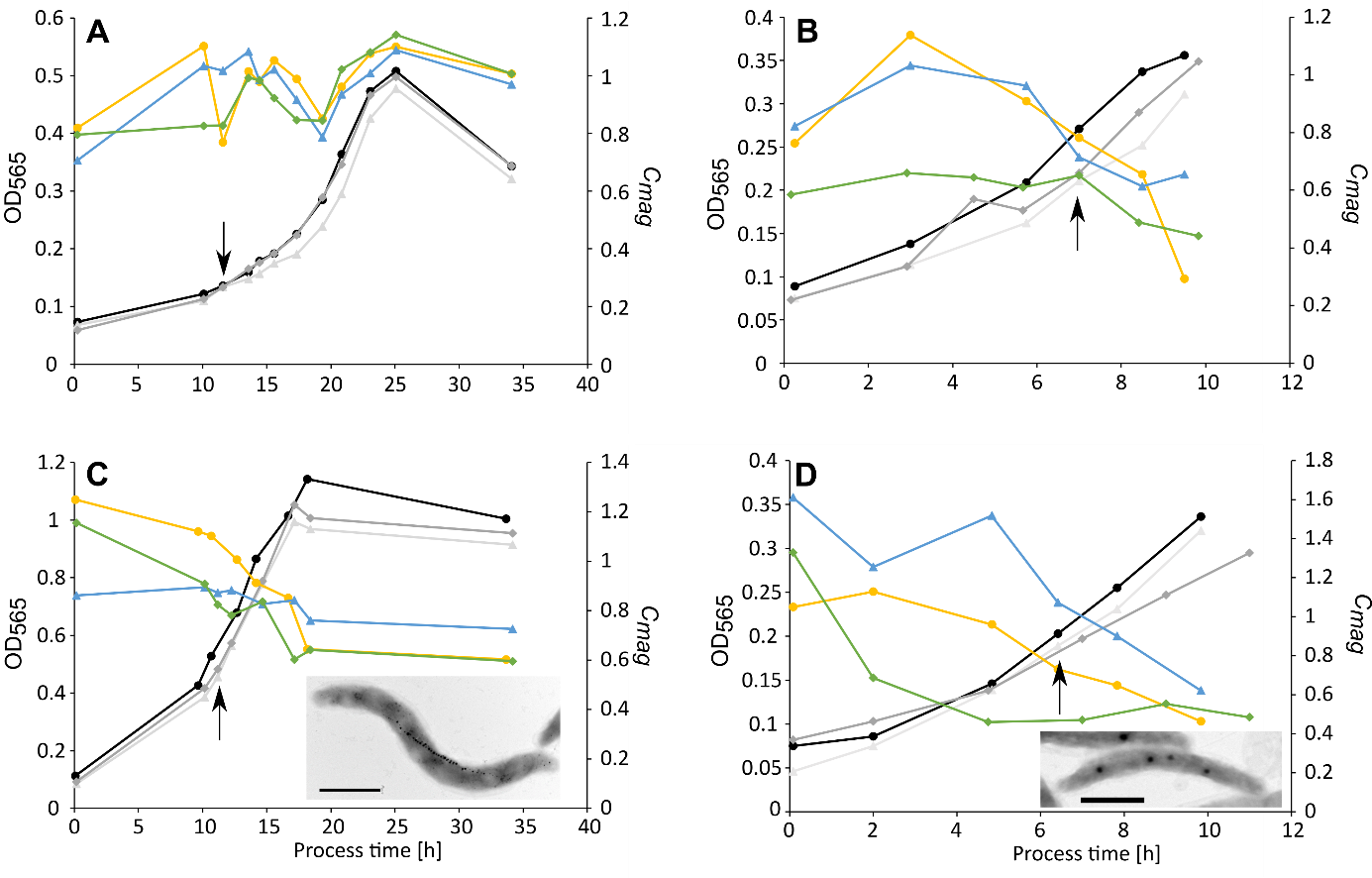


**Figure S1:** Cell growth and magnetic response (C_mag_) under **A**) anoxic (dO_2_ 0%, 10 mM nitrate), B) oxic (dO_2_ 95%, 4 mM ammonium), **C**) microoxic (dO_2_ 1%, 4 mM nitrate), **D**) oxic with nitrate (dO_2_ 95%, 4 mM nitrate) conditions. (Scale bar 1 µm). Growth (black and grey lines) and C_mag_ (colored lines) were depicted for each replicate (circles, diamonds and triangles). The black arrow indicates the sampling timepoint for the RNA-seq experiments.
