# Supplementary material for: The transcriptomic landscape of Magnetospirillum gryphiswaldense during magnetosome biomineralization
Source: BMC Genomics. 2022 Oct 10;23:699. doi: 10.1186/s12864-022-08913-x (PMC9549626; doi:10.1186/s12864-022-08913-x)
Supplement: Supplementary file 3 — Additional file 3: Figure S3. Comparison between promoter motives of TSS located within (inMAI) and outside (exMAI) of the magnetosome island, cultivated under anoxic (0% dO2) and oxic (95% dO2) conditions. The motif logos were created with Weblogo [58]. [file 12864_2022_8913_MOESM3_ESM.docx]

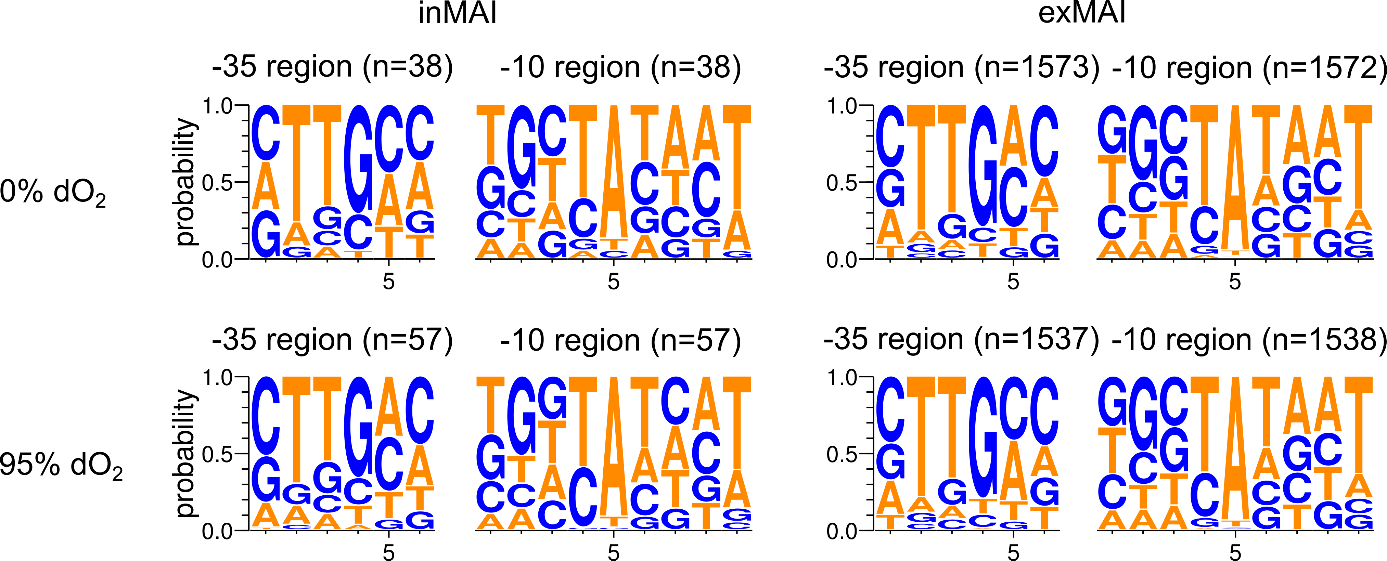


**Figure S3:** Comparison between promoter motives of TSS located within (inMAI) and outside (exMAI) of the magnetosome island, cultivated under anoxic (0% dO_2_) and oxic (95% dO_2_) conditions. The motif logos were created with Weblogo [63].
