# Supplementary material for: The transcriptomic landscape of Magnetospirillum gryphiswaldense during magnetosome biomineralization
Source: BMC Genomics. 2022 Oct 10;23:699. doi: 10.1186/s12864-022-08913-x (PMC9549626; doi:10.1186/s12864-022-08913-x)
Supplement: Supplementary file 4 — Additional file 4: Figure S2. Distribution of classified transcription start sites (TSS) under anoxic (0% dO2) and oxic (95% dO2) conditions in the whole genome (pTSS, primary TSS; asTSS, antisense TSS; iTSS, intragenic TSS; oTSS; other TSS). [file 12864_2022_8913_MOESM4_ESM.docx]

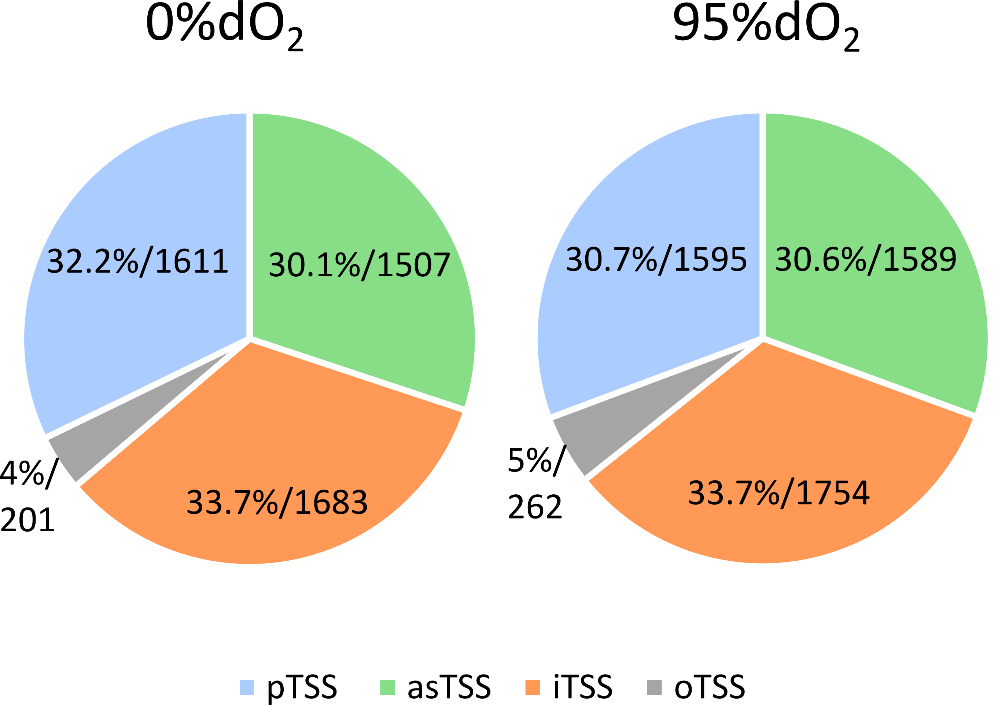


**Figure S 2:** Distribution of classified transcription start sites (TSS) under anoxic (0% dO_2_) and oxic (95% dO_2_) conditions in the whole genome (pTSS, primary TSS; asTSS, antisense TSS; iTSS, intragenic TSS; oTSS; other TSS).
